# Application of MultiStem^®^ Allogeneic Cells for Immunomodulatory Therapy: Clinical Progress and Pre-Clinical Challenges in Prophylaxis for Graft Versus Host Disease

**DOI:** 10.3389/fimmu.2012.00345

**Published:** 2012-11-27

**Authors:** Bart Vaes, Wouter Van’t Hof, Robert Deans, Jef Pinxteren

**Affiliations:** ^1^ReGenesys BVBAHeverlee, Belgium; ^2^Regenerative Medicine, Athersys, Inc.Cleveland, OH, USA

**Keywords:** MultiStem cells, GvHD prophylaxis, regenerative medicine, adherent stem cells, bioreactor

## Abstract

The last decade has seen much progress in adjunctive cell therapy for immune disorders. Both corporate and institutional Phase III studies have been run using mesenchymal stromal cells (MSC) for treatment of Graft versus Host Disease (GvHD), and product approval has been achieved for treatment of pediatric GvHD in Canada and New Zealand (Prochymal^®^; Osiris Therapeutics). This effectiveness has prompted the prophylactic use of adherent stem cells at the time of allogeneic hematopoietic stem cell transplantation (HSCT) to prevent occurrence of GvHD and possibly provide stromal support for hematopoietic recovery. The MultiStem^®^ product is an adult adherent stem cell product derived from bone marrow which has significant clinical exposure. MultiStem cells are currently in phase II clinical studies for treatment of ischemic stroke and ulcerative colitis, with Phase I studies completed in acute myocardial infarction and for GvHD prophylaxis in allogeneic HSCT, demonstrating that MultiStem administration was well tolerated while the incidence and severity of GvHD was reduced. In advancing this clinical approach, it is important to recognize that alternate models exist based on clinical manufacturing strategies. Corporate sponsors exploit the universal donor properties of adherent stem cells and manufacture at large scale, with many products obtained from one or limited donors and used across many patients. In Europe, institutional sponsors often produce allogeneic product in a patient designated context. For this approach, disposable bioreactors producing <10 products/donor in a closed system manner are very well suited. In this review, the use of adherent stem cells for GvHD prophylaxis is summarized and the suitability of disposable bioreactors for MultiStem production is presented, with an emphasis on quality control parameters, which are critical with a multiple donor approach for manufacturing.

## Rationale for Adherent Stem Cells in Prophylaxis

Graft versus Host Disease (GVHD) is a potential life-threatening complication and one of the major limitations of allogeneic hematopoietic stem cell transplantation (HSCT). The complication is thought to be initiated by activation of mature donor T-cells, which are co-infused with the hematopoietic stem cell (HSC) transplant, through recognition of target antigens presented on MHC molecules expressed on antigen presenting cells that reside within host tissue. Upon alloantigen recognition, the co-infused donor T-cells become activated, expand, and induce cytolytic effects that target several organs including skin, gut, and liver.

Current therapies to prevent acute GvHD (aGvHD) make use of pharmacological suppression of T-cell activation, however, such immunomodulatory therapy appears not sufficient to treat a GvHD and it may increase the risk of opportunistic infections (Perales et al., 2007) and disease relapse (Lee et al., 2004). Additional strategies are thus required to improve the response rate to immunosuppression.

The last decades have seen major improvements in stem cell research and the translational application of adult stem cells (Armstrong et al., 2012). This has led to numerous clinical trials to investigate the efficacy of various types of stem cells to treat immune disorders, neurodegenerative and cardiovascular disease, bone and cartilage repair, and type I diabetes (Busch et al., 2011b; Trounson et al., 2011; Penn et al., 2012).

Adherent non-hematopoietic bone marrow-derived stem cells have been demonstrated to reduce proliferation of GvHD patient-derived T-cells (Le Blanc et al., 2004), inhibit alloreactive T-cell responses and support HSC engraftment (Auletta et al., 2010). Their use has therefore gained particular interest to treat and prevent GvHD in patients with hematopoietic malignancies such as acute myeloid or lymphoid leukemia (AML, ALL), chronic myeloid leukemia (CML), or myelodysplasia (MDS).

## MultiStem Cells

Multipotent adult progenitor cells (MAPC) are bone-marrow-derived non-hematopoietic adherent cells that were first described by Jiang et al. (2002). The MultiStem clinical product is based on MAPC isolation and expansion protocols (Boozer et al., 2009). Pre-clinical animal studies have clearly shown therapeutic benefits of MAPC/MultiStem cells by preventing GvHD (Kovacsovics-Bankowski et al., 2009), and improving tissue regeneration and function in cardiovascular and neurological disorders, including acute myocardial infarct, traumatic brain injury, spinal cord injury, and ischemic limb injury (van’t Hof et al., 2007; Aranguren et al., 2008, 2011; Mays et al., 2010; Walker et al., 2010, 2012; Busch et al., 2011a).

MultiStem cells are in Phase II clinical testing for use in treatment of inflammatory bowel diseases (ulcerative colitis), acute myocardial infarct, and ischemic stroke. Safety studies (Phase I clinical trials) using MultiStem as an adjunct in allogeneic bone marrow transplantation for prevention of GvHD (Maziarz et al., 2012) and acute myocardial infarction (AMI) have been completed (Penn et al., 2012). Table 1 summarizes the current status of the MultiStem (pre)clinical trials.

**Table 1:** **Summary of (pre)clinical studies using MultiStem cells**.

Program	Developmental stage clinical trials.gov identifier	Cell administration	Observations	Reference
**INFLAMMATORY AND IMMUNE**				
Ulcerative Colitis	Phase II (in progress) NCT01240915	Single or multiple dose IV	In progress	
HSC transplant/GvHD prevention	Phase I (completed) NCT00677859	1, 5, or 10 million/kg IV, single or repeated dose, adjunctive to HSCT	Doses were safe and tolerated at all doses. Low incidence (11%) of day 100 acute GvHD at 10 million/kg	Maziarz et al. (2012)
Solid organ transplant	Phase I (approved)	2 × 150–600 million cells, first dose transplanted into portal vein at day 1 after transplantation, second dose IV on day 3	In progress	Popp et al. (2011)
	Pre-clinical	2, 4, or 10 million on day-4, or 5 million on days 4 and 0 of allo heart transplant	All doses were pre-clinically safe and well tolerated. Increased long-term pre-clinical allograft protection	Eggenhofer et al. (2011)
**CARDIOVASCULAR**				
Acute myocardial infarction	Phase I (completed) NCT00677222	20 or 100 million via transarterial catheter, 2–5 days after AMI	All doses were safe and well tolerated. Improvement of Ejection Fraction.	Penn et al. (2012)
PVD/PAD/CLI	Pre-clinical	1 million cells intramuscular, 1 day after iliac artery ligation	MAPC induced a more rapid and complete recovery of blood flow than control	Ryu et al. (2011)
**NEUROLOGICAL**				
Ischemic stroke	Phase II (In progress) NCT01436487	IV dose (low/high), 1–2 days after ischemic stroke	All doses were safe and well tolerated	Athersys Press Release, 2 October 2012
Traumatic brain injury	Pre-clinical	2, 10 million cells/kg, IV, 2 + 24 h after injury	MAPC injection has a neuroprotective effect by preserving splenic mass, blood brain barrier integrity, and increasing the brain M2/M1 macrophage ratio	Walker et al. (2010, 2012)
Multiple sclerosis	Pre-clinical	1, 3, or 9 million cells IV after EAE symptom onset	Decreased lesion burden in spinal cord and improved remyelination	Hamilton et al. (2011)
Spinal cord injury	Pre-clinical	200,000 cells transplanted immediately after dorsal column crush injury	Transplantation of MAPC 500 μm caudal to lesion results in prevention of axonal dieback and regeneration of injured axons	Busch et al. (2011a)
Hurler’s syndrome	Pre-clinical	Transplantation into cerebral lateral ventricles	Injection of MultiStem cells in neonatal MPS-I mice reduces the accumulation of GAGs in the brain	Nan et al. (2012)

*Adapted from company website (www.Athersys.com). IV, Intravenous; PVD, peripheral vascular disease; PAD, peripheral arterial disease; CLI, critical limb ischemia; EAE, experimental allergic encephalomyelitis; MPS-I, mucopolysaccharidosis type I; GAG, glycosaminoglycan*.

The therapeutic benefits of MAPC are multimodulatory and have been shown to be caused, at least in part, by their pro-angiogenic effect through trophic support (Aranguren et al., 2007, 2011; Lehman et al., 2012) and their ability to modulate the immune system (Kovacsovics-Bankowski et al., 2009; Walker et al., 2010). In particular the immune-regulatory properties are of paramount importance for GvHD treatment. Human and rodent MAPC are non-immunogenic. The cells lack MHC II expression, and therefore do not induce a proliferative response when co-cultured with allogeneic T-cells (Kovacsovics-Bankowski et al., 2009; Jacobs et al., 2012). These studies showed that MAPC significantly reduce T-cell proliferation when responder T-cells are stimulated with allogeneic irradiated stimulator cells. The study by Kovacsovics–Bankowski furthermore demonstrated the absence of MAPC *in vivo* immunogenicity, since injection of allogeneic Lewis rat MAPC into Buffalo rats failed to prime an anti-Lewis T-cell response as was observed with allogeneic splenocytes. This immuno-privileged nature and capacity of human and rodent MAPC to inhibit T-cell proliferation is of importance for their use in GvHD prophylaxis. A study by Highfill et al. (2009) demonstrated that MAPC had a prophylactic effect on GvHD after intrasplenic injection. Improved animal survival was seen in MAPC treated mice, while reduced CD4+ and CD8+ T-cells in the spleen were observed.

The effect of MAPC on the inhibition of T-cell proliferation in the study by Highfill and coworkers was shown to be dependent on the ability of MAPC to express PGE synthase and the production of prostaglandin E2 (PGE2). Other studies have also shown that MAPC immunosuppression is partially mediated by soluble factors. A role for indoleamine 2,3-dioxygenase has been demonstrated for human and rat MAPC (Kovacsovics-Bankowski et al., 2009; Jacobs et al., 2012), but this was not found in murine MAPC (Highfill et al., 2009). On the contrary, blocking PGE2 activity had no effect on the suppressive effect of human MAPC (Jacobs et al., 2012) indicating that the molecular mechanisms of immunosuppression occur in a species specific manner.

## MultiStem Cells Versus MSC

Mesenchymal stromal cells (MSC) may use similar immunosuppression mechanisms (Gebler et al., 2012) and although MAPC and MSC exert comparable activity in an *in vitro* T-cell suppression assay (Jacobs et al., 2012), it is evident that they are distinct cell types. Both cells are adherent bone marrow-derived stem cells, but due to different culture conditions they adopt different phenotypes (Roobrouck et al., 2011b). The cells express distinct cytokine profiles which may explain the observations that MAPC can induce tube formation by HUVEC cells in *in vitro* assays while MSC lack this pro-angiogenic effect (Lehman et al., 2012). Moreover, MAPC are able to induce functional blood vessels *in vivo* when the cells are implanted in a Matrigel plug with VEGF and bFGF under the skin of nude mice, where vessels induced by MSC appeared leaky (Roobrouck et al., 2011a). This latter study showed by means of transcriptome analysis that MAPC and MSC are clearly distinguishable cells types. In a recent study, intracranial injection of human MAPC and human MSC 2 days after induction of stroke revealed that MAPC had a stronger effect on the attenuation of the inflammatory response and had more potency to promote endogenous tissue regeneration than MSC (Mora-Lee et al., 2012). Thus differences in *in vivo* activity between MAPC and MSC have been described, but it is not clear yet how this relates for instance to the therapeutic activity of these cells in GvHD prophylaxis.

MAPC and MultiStem cells are thus immune-privileged in the currently tested settings, and for MSC low-immunogenicity and an anatomical site-specific immuno-privileged nature have been demonstrated (reviewed by Griffin et al., 2010). Before safe application as an allogeneic cell product to patients, cells need to be isolated, expanded, and quality tested in order to reach sufficient cells with therapeutic activity. This hampers the use of autologous cells for applications where cells are needed immediately, as is the case for stroke for instance. Particularly MAPC and MultiStem cells have the capacity to undergo extensive expansion doublings *in vitro*, which, combined with the immuno-privileged properties, enable an off-the-shelf use for MultiStem cells, with therapeutic product available at the time of need and usable without patient matching.

Pre-clinical animal studies using multipotent MSC in HSCT have shown positive effects on survival benefit and the prevention of GvHD, although contradictory effects are observed depending on the origin of adherent stem cells, timing, and dose of infusion (see reviews by Auletta et al., 2010; Baron and Storb, 2012). Pre-clinical studies have shown safety for intravenous infusion of MultiStem cells and demonstrated that the survival rate in a haploidentical aGvHD rat model increased from less than 20 to 50% in rats that received two MultiStem doses in a prophylactic manner (Kovacsovics-Bankowski et al., 2008, 2009).

Human clinical studies have been conducted to evaluate the safety and efficacy of MSC to treat GvHD. Review of current data show that the application of MSC is safe, but that inconsistent benefit is seen in the treatment of acute and steroid-refractory GvHD (Ball et al., 2008; Auletta et al., 2010; Kebriaei and Robinson, 2011a,b; Baron and Storb, 2012). While these first studies are encouraging and prompt evaluation of optimal dosing strategies for MSC treatment of active clinical GvHD, an equally important strategy is the use of adherent stem cells as adjunct to HSC for prophylaxis of GvHD. To date a limited number of clinical studies have been conducted to investigate co-transplantation of MSC with HSC and the prevention of GvHD. Recently a phase I clinical dose escalation study was finished in which MultiStem cells were administered to adult patients undergoing allogeneic HSCT for the treatment of leukemia and related conditions (Maziarz et al., 2012).

## Clinical Experience Using Adherent Stem Cells as HSCT Co-Transplant

Several studies have evaluated the effect of MSC co-transplantation with HSC on engraftment, safety, and GvHD in pediatric (Ball et al., 2007; Macmillan et al., 2009; Bernardo et al., 2011) and adult patients (Lazarus et al., 2005; Ning et al., 2008; Baron et al., 2010; Table 2). Ball et al. (2007) reported a Phase I/II trial in which 14 children received 1–5 million donor MSC/kg body weight 4 h before peripheral blood stem cell (PBSC) transplantation of HLA-disparate relative donors. No infusion-related toxicity was observed. Graft rejection did not occur in the 14 patients receiving MSC, while seven graft failures were observed in a historic control group of 47 children.

**Table 2:** **Summary of clinical studies using adherent stem cells for GvHD prophylaxis**.

Study	HSCT specifics	Stromal cell therapy	Stromal cell dosing	Observations
Maziarz et al. (2012)	URD, MRD, BM/PB, Adults CSA + MTX, Tac + MTX	Third party, universal donor, GHVD prophylaxis	1, 5, or 10 million/kg, single dose day 2 after HSCT, or 1 or 5 million/kg repeat dose on day 2, 9, and 16, or days 2, 9, 16, 23, and 30 after HSCT	Grade II–IV and III–IV GVHD at Day 100 was 37 and 14%, resp. (*n* = 36). 11% II–IV GVHD and no grade III–IV GVHD and in 10 million/kg group single dose (*n* = 9). Anticipated rates in this population; 47% II–IV and 15% III–IV
Kuzmina et al. (2012)	RD, HSCT, adults CSA, MTX, prednisolone	HSC donor-derived MSC, GVHD treatment	0.9–1.3 million/kg, 19–54 days after HSCT	Grade II–IV aGVHD in 33.3% of control patients and 5.3% in MSC prophylaxis group
Bernardo et al. (2011)	URD, RD, UCB, pediatric CSA + steroids, CSA + MTX	Paternal derived MSC, GVHD prophylaxis	1–3.9 million/kg, single dose at day of HSCT	Reduced grade III–IV GVHD (0%, compared to historic controls 18/8%)
Baron et al. (2010)	URD, PB, adults MMF + Tac	Unrelated MSC, safety of MSC co-transplantation	1–2 million/kg at day of HSCT	Day 100 incidence of grade II–IV was 35%. Cumulative incidence of grade II–IV GVHD was 45%, compared with 56% in historic group
Macmillan et al. (2009)	URD, UCB, pediatric CSA + steroids	Parental MSC, promote engraftment	0.9–5 million/kg at day of HSCT; three patients second dose at day 21	At day 100, cumulative incidence of grade II–IV similar between MSC and historic control (38 versus 22%, *p* = 0.44)
Ning et al. (2008)	RD, BM/PB, adult CSA + MTX	Sibling derived MSC, MSC prophylaxis	0.03–1.53 million/kg at day of HSCT	Grade II–IV was 11.1% in MSC group and 53.3% in non-MSC group. Overall aGVHD incidence was 44.4% in MSC and 73.3% in non-MSC group
Ball et al. (2007)	MRD, PB, pediatric	HSC donor-derived MSC, graft failure	1–5 million/kg single dose at day of HSCT	No graft rejection in patients receiving MSC, 14.8% failure in control group (*p* = 0.14)
Lazarus et al. (2005)	RD, PB/BM, adults CSA + MTX	HSC donor-derived MSC, GVHD prophylaxis	1, 2.5, or 5 million/kg single dose at day of HSCT	Overall, 50% of patients developed aGVHD, at least grade II in 28% of patients. 11 and 4% developed grade III and IV respectively

*URD, unrelated donor; MRD, mismatched related donor; RD, related donor; BM, bone marrow; PB, peripheral blood; UCB, umbilical cord blood; CSA, cyclosporine; MTX, methotrexate; Tac, tacrolimus*.

A decreased incidence of aGvHD was observed in a group of 13 pediatric hematological disorder patients who received paternal HLA-disparate MSC co-transplantation with umbilical cord blood cells (Bernardo et al., 2011). Single dose injections of 1–3.9 million MSC/kg body weight were safe and revealed no significant difference of cumulative graft rejection when compared with a group of 39 historical controls. Grade II–IV aGvHD showed no significant difference between MSC-receiving patients and controls (31 versus 41%, *p *= NS). However, patients in the MSC group did not develop grade III or IV aGvHD, while the incidence of these severe forms in the control groups was 26% (*p* = 0.05). None of the patients developed cGvHD, while 11% were observed in the control (*p* = NS).

Macmillan et al. (2009) reported another Phase I/II clinical trial in pediatric patients receiving MSC co-transplanted with umbilical cord blood transplantation. Eight patients received a dose of 0.9–5 million MSC/kg body weight MCS of haploidentical parental donors, 4 h prior to transplantation of unrelated donor blood cell and three of them were given a second dose at day 21. Three patients developed grade II GvHD, and no patient developed cGvHD. No statistical difference with a historical cohort was observed, but the authors mention a non-significant trend toward improved 3-year survival in the MSC group.

A study by Lazarus et al. (2005) was done on 46 adult patients receiving bone marrow (*n* = 19) or PBSCs (*n* = 27) co-transplanted 1–5 × million/kg MSC from HLA-identical sibling donors. A total of 28% of the patients developed at least grade II aGvHD, while grade III and IV were observed by 11 and 4% respectively. The authors indicate a literature-based percentage of 44–64% for grade III and 12–26% for grade IV, suggesting a benefit of MSC infusion.

Ning et al. (2008) compared patients receiving HLA-identical sibling HSCs from blood or bone marrow without (*n* = 15) or with (*n* = 10) co-transplantation of MSC (0.3–15.3 × 10^5^/kg body weight). Grade II–IV GvHD was developed in 11% of the MSC group and 53% of the non-MSC group. None of the patients in both groups showed grade III–IV aGvHD.

Baron et al. (2010) performed a safety study in which patients were transplanted with PBSCs from HLA-mismatched donors in combination with MSC from third party unrelated donors. Twenty patients were co-infused with PBSC and 1–2 million MSC/kg body weight and compared with historical group of 16 patients treated with unrelated donor PBSC without MSC. In the MSC group, 45% experienced grade II–IV aGvHD and 56% in the control group. Grade IV aGvHD developed in 10% of the MSC group and 19% in the historic group.

The studies by Bernardo, Ning and Baron show that development of aGvHD after HSC transplantation may be reduced after co-injection of MSC. Efficacy of MSC as a therapy for aGvHD has recently also been reported by Kuzmina et al. (2012). In this study the MSC were administered after HSCT at the time of graft activation and GvHD manifestation and the authors showed a significant reduction of the incidence of grade II–IV aGvHD in the group of patients having received MSC. The combined results provide a promising base for adherent stem cells as an adjunct therapy for graft support and GvHD prophylaxis. Still, the number of studies and evaluated patients remain limited, and additional evaluations are essential to determine optimal cell dose, timing, and frequency of administration in achieving maximum clinical benefit.

## MultiStem Therapy for Prophylaxis of Acute GvHD

The primary objective of the clinical Phase I study was to evaluate the safety of MultiStem administration in single dose or as repeat doses to patients receiving allogeneic HSCT (Maziarz et al., 2012). A total of 36 patients was treated with MultiStem, 18 each in the single dose arm (1, 5, and 10 million cells/kg on day 2 after transplant) or the repeated dose arm (1 or 5 million/kg on days 2, 9, and 16 (3 weekly doses), or 5 million/kg on days 2, 9, 16, 23, and 30 (5 weekly doses).

The study demonstrated that MultiStem therapy was well tolerated in both the single infusion and repeat infusion arms and also suggested that the therapy may provide benefit to recipients of allogeneic HSCT, such as reducing the incidence and severity of GvHD, as compared to historical clinical experience (Ratanatharathorn et al., 1998; Nash et al., 2000; Anasetti et al., 2011). The majority of patients participating in the study received transplants from unrelated donors (19 of 36), and nearly all of the patients received PBSC transplants (34 of 36), both of which are associated with a higher risk of GvHD. Importantly, all patients experienced successful neutrophil engraftment (median time of engraftment 15 days), and 86% of patients experienced successful platelet engraftment (median time of engraftment 16 days) which compares favorably to historical clinical experience for this patient population supporting a positive impact on blood and immune system recovery. Relative to the published experience for this specific patient population (Ratanatharathorn et al., 1998; Nash et al., 2000; Anasetti et al., 2011), there was a substantial reduction in aGvHD incidence after administration of the highest single dose of 10 million MultiStem cells/kg, i.e., 11% grade II–IV GvHD, and 0% grade III–IV GvHD, versus 45–70 and 15–20%, respectively. There appeared to be a trend in dose response relationship, with patients receiving the highest single dose of MultiStem cells having a 33% lower absolute incidence of aGvHD relative to patients who received a single low or medium dose, and patients receiving once weekly dosing of the medium dose through the first 30 days having reduced GvHD incidence relative to single or weekly dosing over the first 2 weeks post-transplant. Finally, relapse-free survival rate at 100 days and infection-related complications over the first 100 days were favorable relative to historical clinical experience, consistent with the positive effect on engraftment rates.

## Challenges in Stem Cell Therapeutic Product Development

Review of the stromal cell co-transplant and GvHD prophylaxis studies summarized above reveal an important limitation to the complete and optimal use of MSC as an effective therapy. Three of the studies could not be performed as planned because of insufficient availability of MSC at the time of transplantation (Lazarus et al., 2005; Ning et al., 2008; Macmillan et al., 2009). As a result, patients were not injected, not given a repeat dose, or given lower doses of MSC. This illustrates that the use of donor-related MSC is hampered by the limited proliferative capacity of these cells and/or sub-optimal cell expansion protocols or procedures. For efficient therapeutic application in the clinic, most of these limitations would be overcome by use of an allogeneic of-the-shelf stem cells product that is expanded to large scale with consistency in yields and quality.

To illustrate how the cell dose requirements for clinical studies impact the associated expansion and quality control needs we will detail the MultiStem study as a paradigm. For the completion of the entire MultiStem GvHD study, a total of 35 billion cells were injected, all of which were derived from expansions of seed-stock obtained from a single donor. Current MultiStem production units contain a surplus of cells, and consequently, over 50 billion cells were required for this trial. Of course, a multiplicity of cells will be needed for future trials and new manufacture procedures are required to produce the cells in a safe and cost-effective manner. Current process development efforts focus on the optimization of stem cell manufacturing in order to achieve a consistent and safe product for off-the-shelf use.

## MultiStem Manufacturing

One of the most advantageous features of MultiStem cells is the proliferative capacity, and cells can undergo more than 60 population doublings (PD) before senescence. The extensive proliferation capacity allows creation of a master and working cell bank as production intermediates. The current manufacturing strategy is based on clinical doses generated at about PD28 (master cell bank campaign) or PD38 (working cell bank campaign) that allows for the production of >100,000 clinical doses from a single donor.

MultiStem clinical production is currently performed by a contract manufacturing organization (Lonza) for creation of master cell banks and for production campaigns starting from those banks. A production run typically generates 40–50 clinical doses. The clinical dose varies according to indication, but current production units contain 180 million cells. The cells are cryopreserved and stored in a mixture of PlasmaLyte, i.e., an isotonic solution that mimics human plasma electrolytes, pH and osmolality (Baxter), DMSO, and human serum albumin. Each production run is tested for adventitious agents such as sterility, mycoplasma, and endotoxin. The product is also tested to show a normal karyotype. Only after completion and validation of all tests, the product is released from the contract manufacturer and stored for sites that take part in MultiStem clinical trials. Currently, the product has a validated shelf-life of 5 years.

## MultiStem Cell Expansion in a Hollow-Fiber Bioreactor

The Quantum Cell Expansion System (TerumoBCT) is being explored as an alternate platform for larger scale cell culture. This instrument was developed for *ex vivo* expansion of stem cells using a hollow-fiber bioreactor (Antwiler et al., 2009). The functionally closed automated culture system is comprised of a disposable synthetic hollow-fiber bioreactor of 2.1 m^2^ surface area connected to a sterile closed-loop, computer-controlled media perfusion platform and gas exchangers. In addition, the system contains sterile closed sample ports by which fluid samples can be taken during expansion in order to monitor expansion and estimate the appropriate moment of harvest.

The Quantum system has been tested to optimize the complete workflow of MultiStem culture in a two-step procedure of stem cell isolation from bone marrow and subsequent expansion up to the scale of clinical dose. During the first step, whole bone marrow is loaded onto a bioreactor and maintained for 10 days, yielding 1 × 10^7^ MultiStem cells. These cells are loaded onto a new bioreactor and expanded to 1 × 10^9^ cells within a period of 6 days. Thus five doses of 180 million cells are obtained by using two consecutive runs on this bioreactor. This encourages the further exploration of this system to upscale MultiStem batches that are sufficient for clinical studies.

**Figure 1:**
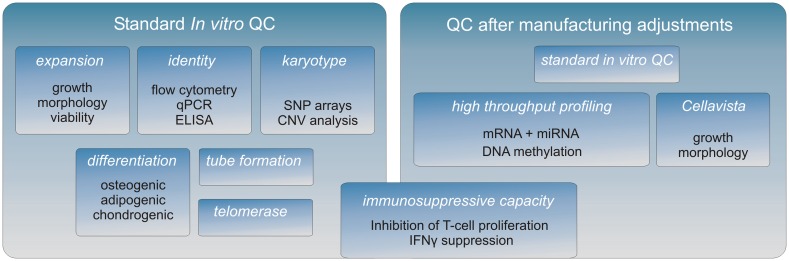
**The MultiStem QC pipeline**. A full characterization of the MultiStem product is being conducted after each important adjustment of the manufacturing procedure. First, a standard QC is performed to establish MultiStem growth and typical stem cell properties (left panel). Subsequently, high throughput screens are performed to investigate the molecular phenotype of MultiStem (right panel). The Cellavista image-based platform (Roche) is used to study various morphological aspects of different cell cultures. Genome-wide molecular phenotype analyses are carried out on different platforms including array technology, PCR-based screening, and next-generation sequencing (NGS). Combining these “omics” data facilitates on the one hand the identification of unique MultiStem features, while on the other hand the retention of the molecular identity after applying alternative culturing methodologies can be validated. For MultiStem equivalency testing, the immunosuppressive capacity is evaluated by two assays: one is based on inhibition of T-cell proliferation and the other is based on the corresponding reduction of IFNγ secretion by T-cells.

A crucial aspect of the research is to confirm by means of *in vitro* cell equivalency testing that the expanded cells are of consistent high quality and that cellular features that relate to *in vivo* function are maintained after manufacture adjustments (Figure 1).

## MultiStem Quality Control and Equivalency Testing

In advancing toward a final optimized manufacturing process, modifications of the MultiStem manufacturing process are extensively controlled in order to keep a consistent quality of the product. A panel of cell assays has been developed that allows for MultiStem QC testing in a tiered testing strategy (Figure 1). First, MultiStem identity is measured by marker gene and protein expression analysis by means of QPCR, ELISA, and flow cytometry. For MultiStem batches that pass these criteria, multilineage differentiation assays that are typically associated with stem cells from mesenchymal origin are performed. Osteogenic, adipogenic, and chondrogenic differentiation are measured by means of *in situ* cytochemistry and specific gene induction profiles associated with the corresponding cell types. As indicated above, one of the MultiStem product’s mode of action *in vivo* has been shown to be based on pro-angiogenic activity, which is supported by an *in vitro* tube formation assay correlated with *in vivo* angiogenic activity and cytokine expression (Lehman et al., 2012).

Given the indications that inhibition of T-cell proliferation is a major contributor to immune suppression by stromal cells, we consider *in vitro* immune suppression and potency assays as highly important in our QC in order to guarantee a consistently safe product for treatment of GvHD and other disorders. With the purpose of application in GvHD and other disorders in which immunosuppression by MultiStem cells is critical, our QC emphasizes on *in vitro* assays that reflect such suppression (Kovacsovics-Bankowski et al., 2009). An *in vitro* criterion that is often used to assess the immunosuppressive capacity of stem cells is their inhibitory effect on the proliferation of activated T-cells. In our QC we use two different standardized assays to quantify MultiStem immunosuppression. One of the assays directly measures the inhibition of T-cell proliferation in a co-culture model of MultiStem cells and responder T-cells that are activated by CD3/CD28 or PBMC (Jacobs et al., 2012) while the other assay quantifies interferon gamma (IFNγ) which is secreted by activated T-cells.

In the context of biosafety, a normal karyotype is demonstrated by means of copy number variation (CNV) analysis of MultiStem cells and donor-derived non-expanded mononuclear cells on SNP arrays and the data is analyzed for genomic insertions or deletions at a resolution of 50 kb.

## Epigenetic Screens for Cell Equivalency Testing

Our current QC pipeline is sufficient to determine MultiStem identity and lot release assays for early to mid-stage clinical studies. However, it is anticipated that for late stage clinical trials (Phase III) and product release, more stringent quality controls are required by the regulatory organizations FDA and EMA, particularly in terms of potency and comparability following process improvements. The QC pipeline is being extended with various genome-wide screening methods to comprehensively characterize the molecular phenotype of our product. Transcriptome analysis is already implemented as a powerful tool in cell comparability testing, and we currently explore emerging epigenetic analysis tools that on the one hand identify robust MultiStem markers and on the other hand provide insight in the mechanisms underlying MultiStem function.

One of the epigenetic tools to investigate MultiStem identity and comparability is miRNA screening, since miRNA profiles determine the identity of stem cells (Chen et al., 2007) and distinguish between embryonic or adult stem cells, as well as between the adult stem cells MAPC and MSC (Aranda et al., 2009). Epigenetic modifications such as DNA methylation or histone modification can influence the function of the associated genes. As a consequence, stem cell identity is related to the epigenetic profile and differentiation capacity is determined by epigenetic components, including DNA methylation and histone modification (Bloushtain-Qimron et al., 2009; Weishaupt et al., 2010). Epigenetic processes can become altered by cell culture processes, since methylation of genes related to differentiation can change during *in vitro* passaging (Bork et al., 2010), while the maintenance of unmethylated regions appears serum-dependent (Dahl et al., 2008). This underscores that monitoring of epigenetic processes may lead to a breakthrough in therapeutic stem cell manufacturing development. Recently, a next-generation sequencing methodology was started to map cytosine methylated regions in MultiStem cells and to explore the possibility of identifying DNA methylation markers. An additional epigenetic assay that is under development to distinguish MultiStem cells from other adherent stromal cells such as MSC is based on telomere biology, an important predictor for proliferative capacity, and it was recently shown that MultiStem telomerase activity is much higher than that of MSC (Boozer et al., 2009).

Altogether, these assays will serve as controls for epigenetic stability, and the product uniqueness and consistency, in particular after modification of the MultiStem manufacture procedure. Application of these QC assays confirmed that characteristics of MultiStem cells harvested from the Quantum Cell Expansion System were maintained compared to those under standard cell culture conditions (data not shown). All QC assays performed thus far indicate successful MultiStem expansion in this bioreactor format, with significant advantages in air-handling requirements and reductions in labor.

## Perspective

Currently for the stromal cell therapy field as a whole, and for MultiStem in particular, the development is still mainly in the pre-clinical and early and mid clinical stages, during which safety and dose effects are being evaluated. The optimal dosing strategy for stromal cells is considered to be the composite of optimal individual dose level/administration and minimal number of administrations required to fully cover therapeutic opportunity windows. E.g., in the case of GvHD prophylaxis, the therapeutic window covers 30–45 days after allo HSCT. Ideally, clear efficacy is observed after infusion of a single dose level of cell product, but this has not consistently been the experience in pre-clinical or clinical evaluations (Table 2). As a consequence, current manufacture strategies are based on the anticipated need to repeat infuse medium to high dose levels (5–10 million cells/kg of bodyweight) in order to observe efficacy. This equates roughly to 400 million to 1 billion cells/infusion, or >1 billion cells/patient for repeat administration, which levels are outside of the range for MSC production from individual donors on a consistent scale. This is especially the case in context of anticipated late stage Phase III clinical studies with large numbers of subjects (>100). However, for the MultiStem product these cell requirements can feasibly be covered with material from individual donors by using a staged expansion and banking approach based on the extensive expansion capacity of the MultiStem platform.

In all, the early clinical observations indicate that the class of stromal stem cells can be safely infused via single or repeat dose regimens in humans without long-term complications. There are no apparent disadvantages of MultiStem *per se*, compared to MSC. Still, continued clinical evaluation and scrutiny will be required to address the still fairly limited experience with immune sensitization as a consequence of repeat dosing of allogeneic product, or long-term risk of ectopic tissue formation, especially in immune-compromised subjects. One remaining shared disadvantage in current use of MultiStem and MSC is the use of FBS for product manufacture and this will be a major area of need in the development of next-generation cell therapy products. Immune responses have been detected against serum components on the stromal cells, but no significant alloantibody production has been reported (Spees et al., 2004; Sundin et al., 2007). Completion of a serum-free workflow will be beneficial because of limited serum availability, batch-to-batch differences, the possibility of adventitious pathogens and ethical considerations. It is anticipated that Phase III studies using MultiStem in several clinical indications will have integrated serum-free media formulation and production in a closed bioreactor format.

## Conflict of Interest Statement

The authors declare that the research was conducted in the absence of any commercial or financial relationships that could be construed as a potential conflict of interest.
